# Expanded Programme on Immunization (EPI): A Legacy of 50 Years and the Road Ahead

**DOI:** 10.3390/vaccines13060649

**Published:** 2025-06-17

**Authors:** Imran Mirza, Ephrem Tekle Lemango, Ann Lindstrand

**Affiliations:** 1 United Nations Children Fund (UNICEF) Headquarter, New York, NY 10017, USA; elemango@unicef.org; 2 Essential Programme on Immunization Unit, Immunization Vaccines & Biologicals Department, World Health Organization (WHO), 1202 Geneva, Switzerland; lindstranda@who.int

## 1. A Global Health Triumph

Throughout history, disease and epidemics have shaped human survival. However, the introduction of vaccines transformed this narrative, offering powerful protection against deadly infections. After World War II, Europe faced dire living conditions that fueled the spread of diseases like tuberculosis (TB), especially among children. In response, health workers deployed the BCG vaccine widely, reaching over 11 million children by 1950 [1]. This milestone, as well as the successful smallpox eradication program, marked a turning point in public health, laying the foundation for global immunization efforts that would go on to protect millions of children from other life-threatening diseases such as measles, diphtheria, and pneumonia.

Fifty years ago, the World Health Organization (WHO) launched [2] the Expanded Programme on Immunization (EPI) to ensure that children across the world, regardless of their socioeconomic status, had access to life-saving vaccines. What began as an ambitious initiative to combat six major infectious diseases—diphtheria, pertussis, tetanus, measles, poliomyelitis, and tuberculosis—has since evolved into one of the most significant public health interventions in history [3]. A recent study [4] showed that every dollar spent on immunization during 2011–2020 produced an average return of up to USD 26 across a child’s lifetime, thanks to savings on health care expenditure and lost revenues due to illness. Today, rebranded as the Essential Programme on Immunization (EPI), the initiative has expanded its reach, integrated newer vaccines, and adapted to emerging health challenges. By preventing at least 154 million deaths since 1974, the program has dramatically reshaped the trajectory of global health, contributing to infant survival, disease eradication efforts, and broader health care infrastructure development [3].

As we commemorate the 50th anniversary of EPI, it is important to take stock of the gains in public health this flagship program has accomplished (Table 1), although this global health architecture is also currently under acute financial threat. This Editorial takes an in-depth look at EPI’s evolution, achievements, challenges, and the future course of immunization efforts worldwide.

## 2. The Evolution of EPI: Expanding Immunization Frontiers

### 2.1. The Early Years (1974–1990): Laying the Foundation

The first 15 years of the Expanded Programme on Immunization focused on establishing standardized immunization schedules and ensuring access to vaccines in low-income countries through standardized vaccination programs and outreach campaigns. Vaccination coverage was alarmingly low, with only about 5% of children in low-income countries and 21% worldwide receiving basic vaccines in 1974.

The turning point came in 1980 with the certification of the global eradication of smallpox, proving that a well-coordinated vaccination campaign could wipe out a deadly disease. This success laid the groundwork for more ambitious vaccination efforts, particularly the push for polio eradication in the late 1980s.

During this phase, UNICEF, WHO, and global donors played a critical role in advocacy for immunization, funding vaccine procurement, training health care workers, and strengthening cold chain logistics to reach the most vulnerable populations.

From 1982 to 1990, coverage increased with speed globally, from around 20 to 76% of DTP3 [5], fueled by the ‘Child Survival and Development Revolution’ initiated by Jim Grant in UNICEF [6] and jointly supported by WHO.

However, a major challenge during this phase was the lack of robust cold chain systems that can keep vaccines potent at the last mile, especially in low-resource settings where electricity and refrigeration were unreliable.

### 2.2. The Growth Phase (1990–2010): Introduction of New Vaccines and Cold Chain Innovations

As global immunization coverage increased more slowly in the 1990s—reaching over 81% for diphtheria, pertussis, and tetanus (DPT3) vaccines by 2008—the EPI expanded its scope [7]. Several key milestones defined this period, such as the introduction of hepatitis B, Hemophilus influenza type b (Hib), and pneumococcal conjugate vaccines (PCV) into routine immunization schedules.

The launch of GAVI, the Vaccine Alliance, in 2000 played a transformative role in funding vaccine access for lower-income countries (LIC). Since then, the GAVI Alliance, initially formed by WHO, UNICEF, BMGF, and the World Bank and later expanding to many more partners, has helped countries to reach over 1 billion children with lifesaving vaccines and expanded the number of vaccines available for low- and middle-income countries (LMIC) from protection of 7 to 20 infectious diseases.

Innovations and an expanded use of new vaccine delivery technologies happened during the phase, such as auto-disable syringes and better cold chain storage solutions, ensuring improved vaccine safety and efficiency. Another key achievement in this phase was the introduction of vaccine vial monitors (VVMs)—a critical innovation allowing health care workers to detect heat exposure in vaccines—as well as an expansion of cold chain storage capacity, including solar-powered refrigerators, enabling better vaccine storage in rural and off-grid areas [8].

By the early 2000s, innovations in logistics, temperature monitoring, and transportation had significantly reduced vaccine wastage and improved reach to last-mile populations. By 2010, it resulted in over 100 million infants being vaccinated annually, preventing millions of deaths and hospitalizations due to vaccine-preventable diseases, and bringing global DTP3 vaccine coverage to 83% [9].

### 2.3. The Current Era (2010–2024): Addressing New Challenges and Expanding Immunization Technologies

Over the last decade, immunization programs have faced both remarkable achievements and unprecedented challenges—the EPI has had to navigate new obstacles such as pandemic/outbreak-related disruptions (e.g., Ebola, H1N1 influenza, Zika, COVID-19, etc.), vaccine hesitancy, and the need for advanced cold chain solutions for new-generation vaccines.

Some of the major achievements in this phase were the introduction of new vaccines such as rotavirus, HPV (human papillomavirus), and malaria vaccines [10] in many national immunization programs to significantly reduce mortality among women and child mortality due to diarrheal disease and prevent cervical cancer and the near eradication of wild poliovirus in most countries, with only a handful of endemic cases remaining. There were advances in remote temperature monitoring (RTM) technologies, allowing real-time tracking of vaccine storage conditions. A new focus became the integration of immunization into broader primary health care systems, linking vaccination to maternal and child health services. In this era, there was a significant decline in under-five mortality rates, largely due to immunization against pneumonia, diarrhea, and measles.

The COVID-19 pandemic (2020–2023) severely disrupted routine immunization services, leading to backslides in coverage, particularly in measles and polio vaccinations [11]. Reasons for this included redirection of the health workforce towards the COVID-19 response, lack of health care workers, lockdowns leading to logistical challenges, and fear of transmission in health care settings. However, despite the impact on routine immunization coverage, established immunization programs played a vital role in the COVID-19 response by providing the infrastructure, workforce, and systems needed to rapidly scale up vaccine delivery, particularly in low- and middle-income countries. Their ability to vaccinate millions, several times more than in the years before, efficiently highlights their adaptability and positions them as key tools for managing future global health emergencies such as MERS, SARS, mpox, and Ebola, reinforcing their importance in global health security. Cold chain requirements for mRNA vaccines posed a new challenge during the pandemic, requiring ultra-cold storage solutions.

Global DTP3 coverage, however, was stagnating around 85% [12], with a severe decrease during the pandemic years 2020–2023.

## 3. Setbacks and Emerging Challenges

Decreased acceptance or even vaccine hesitancy—driven by misinformation, distrust in institutions, and anti-vaccine movements—has become a major public health concern and, together with the coverage decrease during the pandemic, has led to the resurgence of vaccine-preventable diseases like measles and polio. The COVID-19 pandemic had a heterogeneous impact on vaccine confidence: while adult uptake of influenza, pneumococcal, and COVID-19 booster vaccines increased in several contexts [13], perceptions of childhood vaccine importance declined in multiple sub-Saharan African countries [14].

Unvaccinated and under-vaccinated children are accumulating in conflict zones, displacement crises, and areas with weak health infrastructure. Up to half of the so-called zero-dose children live in conflict and humanitarian settings where insecurities hinder vaccine delivery in fragile settings such as Afghanistan, Syria, Sudan, and Yemen [15,16].

The increasing cost of newer vaccines has raised concerns about financial sustainability and equitable access, especially in middle-income countries transitioning out of Gavi support.

When EPI started, there were six antigens included in the program, including diphtheria, tetanus, pertussis, tuberculosis, polio, and measles. EPI now handles universally recommended vaccines against 13 antigens, and there are vaccines covering more than 30 antigens and many more in the pipeline. This ever-expanding number of vaccines poses delivery challenges in countries with health care worker gaps and weak health care systems. In addition, particularly in Africa, the yearly birth cohort is expanding and requires scaling up of PHC services [17,18].

Many children remain unvaccinated because they live in areas with little or no access to primary health care—a comprehensive approach that encompasses health promotion, disease prevention, and treatment. While vaccine campaigns are effective in reaching these children, they are inherently time-bound and cannot provide consistent, ongoing services. To ensure all children are vaccinated in a reliable and sustainable way, it is crucial to integrate childhood immunization into strengthened primary health care systems.

In 2025, global health faced a major setback when key donors imposed a budget freeze, resulting in the termination of numerous health programs and tighter restrictions on those that remained [19]. This event exposed the fragility of health systems in the Global South, which remain heavily dependent on external aid. In response, low- and middle-income countries (LMICs) are being urged to reassess their financial dependencies and develop regional strategies for sustainable health financing and international collaboration. The crisis has reinforced the importance of South–South cooperation and the sharing of knowledge and best practices among LMICs. A University of Washington report [20] warned that the funding freeze could trigger a global health crisis, while the World Economic Forum emphasized [21] the urgent need for countries to invest in domestic health infrastructure, enhance local capacity, and build greater resilience and health sovereignty.

## 4. The Road Ahead: The Next 50 Years of Immunization

### 4.1. Tackling Vaccine Hesitancy and Misinformation

As immunization programs transition into a new era characterized by scientific advancement and expanded life-course vaccination platforms, one of the most enduring impediments to achieving high coverage is behavioral rather than biological. Despite significant improvements in global vaccine supply, under-vaccination—driven by both supply- and demand-side determinants, including vaccine hesitancy—remains a critical public health challenge. The pandemic underscored the profound influence of social and psychological factors on vaccine uptake. Trust, particularly in institutions and interpersonal relationships, emerged as a decisive factor. A cross-national study [22] involving 177 countries found that high levels of governmental and interpersonal trust were the only pandemic preparedness indicators consistently associated with increased COVID-19 vaccine coverage. Conversely, belief in COVID-19-related misinformation was strongly correlated with hesitancy in over 40 countries, with misinformation exposure being particularly pervasive in low- and middle-income countries (LMICs) [23].

Addressing under-vaccination requires a nuanced understanding of vaccine acceptance within the broader context of the caregiver’s journey. Structural barriers—such as geographic inaccessibility, inadequate service integration, and opportunity costs associated with vaccination—often exert a greater influence on caregiver behavior than concerns related to vaccine safety or efficacy. In many cases, non-vaccination may reflect informational or logistical constraints rather than attitudinal opposition, particularly among caregivers who are unaware of where, when, or how to vaccinate. These access-related impediments disproportionately affect women [24], who frequently bear the burden of caregiving alongside structural and social constraints.

A paradigmatic shift from reactive to proactive, context-specific, and well-resourced demand generation strategies is required. Trust-building must be institutionalized as a core component of routine health systems strengthening. This includes systematic engagement with communities, the provision of equitable and high-quality health services, and the empowerment of trusted local actors. Ghana’s national response to vaccine misinformation during the COVID-19 pandemic—anchored by a multisectoral task force and comprehensive training for health promotion officers in misinformation management—provides a replicable model for institutionalizing trust-building mechanisms within national health programs [25]. Effective demand strategies must also be grounded in behavioral and social insights, using, for example, the WHO-developed Behavioral Science and Demand Tool (BeSD). Routine social listening and community-level feedback mechanisms can ensure that communication efforts are contextually resonant. For example, a global cross-sectoral initiative deployed behavioral science-informed messaging to over 100 million individuals, testing variants in message content, tone, and messenger identity to optimize vaccine-related outcomes [26]. Similarly, the UNICEF-supported “Cranky Uncle Vaccine” initiative [27], a gamified mobile platform employing inoculation theory and critical thinking, has demonstrated success in improving misinformation recognition and intent to vaccinate in five countries across Africa and Asia [28].

Health care workers continue to be among the most trusted sources of vaccine information yet often lack the competencies or confidence to address caregiver concerns effectively. Evidence from six countries in Europe and Central Asia indicates that perceived vaccine safety, strong provider recommendations, and frequent health consultations are key determinants of timely childhood immunization [29]. Training programs must therefore integrate interpersonal communication (IPC) skills within broader clinical and service quality improvement efforts to maximize their impact. Community engagement, grounded in respect, co-creation, and sustained dialog, is foundational to equitable immunization coverage. In Remo North, Nigeria [30], a participatory action research approach involving community leaders, frontline providers, and local authorities led to a 30-percentage point increase in full immunization coverage among children aged over nine months. Such models enhance both the legitimacy of health interventions and the responsiveness of systems to local needs.

Looking ahead, addressing vaccine hesitancy and under-vaccination will require a systems-level transformation that integrates trust-building, real-time social and behavioral insights, digital innovation, and equity-centered community engagement. Immunization programs must move beyond top–down messaging to embrace participatory, adaptive strategies that reflect and respond to the lived realities of the populations they serve. Only through such approaches can vaccination be re-established as a shared social norm and a cornerstone of global public health.

### 4.2. Investing in New Vaccine Development

The future of immunization hinges on sustained investment in vaccine research and development (R&D) to address both longstanding and emerging infectious disease threats. The COVID-19 pandemic catalyzed a historic surge in global investment, accelerating the development and regulatory approval of new vaccine platforms—particularly mRNA-based technologies—which have since opened the door for faster, more scalable vaccine production for other diseases [31]. These shifts have not only enhanced pandemic preparedness but also unlocked new potential for combating persistent global health challenges.

Building on this momentum, the next 50 years will see an expanded pipeline targeting high-burden diseases like tuberculosis, malaria, respiratory syncytial virus (RSV), and rotavirus. Several high-priority vaccines are on the horizon. Universal influenza vaccines are under development to provide broader and longer-lasting protection against seasonal and pandemic strains. Similarly, more effective tuberculosis (TB) vaccines aim to replace or enhance the Bacillus Calmette–Guérin (BCG) vaccine, which has limited efficacy against adult pulmonary TB. With TB remaining one of the top infectious disease killers globally, novel vaccine candidates like M72/AS01E show promise in late-stage trials [32]. Furthermore, mRNA technology, validated during the COVID-19 response, is now being applied to a range of emerging and neglected diseases, including Zika, Lassa fever, HIV, and antimicrobial-resistant infections. The adaptability and scalability of mRNA platforms offer hope for more rapid, targeted vaccine responses in the future.

To expand accessibility and address delivery bottlenecks, novel vaccine formats such as mucosal vaccines (e.g., nasal sprays and oral tablets) and needle-free technologies like microneedle patches [33] are being developed. These innovations enhance immunogenicity, reduce cold chain dependencies, and facilitate administration in hard-to-reach settings.

In parallel, global efforts are strengthening local vaccine R&D and manufacturing capacity. Less than 1% of vaccines used in Africa are produced on the continent [34]—a stark inequity that the African Union seeks to remedy by targeting 60% local production by 2040. To that end, new mRNA vaccine facilities are being established across Africa, Asia, and Latin America, supported by global initiatives like the WHO mRNA Technology Transfer Hub and the Developing Countries Vaccine Manufacturers Network (DCVMN) [35]. Building on lessons from the pandemic, global partners must continue to support vaccine innovation and equitable access. Prioritizing the development of next-generation vaccines, strengthening local manufacturing, and adopting delivery innovations will be key to ensuring that every child, no matter where they live, can benefit from life-saving immunizations.

Recent breakthroughs in artificial intelligence and nanotechnology are set to transform vaccine development and delivery. AlphaFold 3, an advanced AI tool, accelerates vaccine target identification by accurately predicting molecular interactions vital for immune responses, significantly aiding research into complex diseases like malaria [36]. Simultaneously, nanotechnology innovations, including lipid nanoparticles used in mRNA COVID-19 vaccines and emerging layer-by-layer nano-delivery systems, offer precise, scalable methods for vaccine delivery and controlled antigen release [37]. Together, these technologies pave the way for faster, more adaptable, and more effective vaccines to meet evolving global health challenges.

Together, these scientific and structural investments will define the immunization agenda for decades to come—enabling broader protection, faster pandemic responses, and equitable access to life-saving vaccines for all.

### 4.3. Expanding Life Course Immunization

As we look toward the future of global immunization, transitioning from a child-centric paradigm to a life course approach will be imperative to meet the evolving health needs of populations and to realize the full potential of immunization in achieving universal health coverage (UHC), health equity, and pandemic preparedness. While childhood immunization has historically driven dramatic reductions in vaccine-preventable diseases (VPDs), the growing epidemiological burden among adolescents, adults, and older persons signals a pressing need to expand protection across all age groups.

Demographic transitions, including rapid aging in many regions, have created new challenges. By 2050, the number of people aged 60 and older will exceed 2 billion globally [38], many of whom are at heightened risk of VPDs such as influenza, pneumococcal disease, shingles, and respiratory syncytial virus (RSV). Nevertheless, few countries have established robust immunization platforms for older adults. Without integration of routine adult vaccination into primary health care, countries risk increased hospitalizations, mortality, and economic strain due to preventable disease outbreaks.

The adolescent and adult age groups have also been neglected in most national immunization schedules. HPV vaccination, which can prevent cervical cancer, remains underutilized in many low- and middle-income countries (LMICs) [39], and uptake of vaccines for tetanus, hepatitis B, and COVID-19 among adults is often inconsistent. Adolescents and adults represent both a vulnerable group and an untapped opportunity. Adolescents, for instance, are accessible through school systems, reproductive health services, and digital technologies, making them a strategic target for catch-up vaccinations and building trust in health services. Similarly, adults engaged through antenatal care, workplaces, and non-communicable disease (NCD) clinics can be reached with maternal, influenza, and COVID-19 vaccines, enhancing disease prevention and improving continuity of care [40].

Importantly, life course immunization is not only a public health imperative but also an economic and social investment. Studies have shown that expanding vaccination beyond childhood reduces productivity losses, prevents health care costs associated with preventable illnesses, and fosters healthier, longer-living populations [41]. It also enhances pandemic preparedness by maintaining immunity across the broader population, minimizing the impact of infectious disease outbreaks on health systems and economies. Adult and elderly immunization played a crucial role in reducing severe COVID-19 outcomes and helped maintain the continuity of essential services during the pandemic—an experience that underscores the value of this approach.

Operationalizing life course immunization will require structural shifts in policy, financing, and service delivery. Governments must update national immunization plans and essential benefits packages to include vaccines for all age groups. Health systems need to strengthen delivery platforms beyond childhood, integrating vaccination into maternal health, occupational health, geriatric care, and chronic disease services. Community health workers must be trained and equipped to deliver age-appropriate vaccines in outreach and home-based settings. Furthermore, digital health tools, such as electronic immunization registries, can improve tracking and targeting of populations across the lifespan, enabling better forecasting, follow-up, and impact assessment.

Equally crucial is securing sustainable financing and strong political will. Life course immunization demands long-term investment and cross-sectoral collaboration. Leveraging existing platforms—such as school health programs, social protection schemes, and mobile health campaigns—can enhance efficiency and reach. Countries should also adopt a whole-of-society approach by involving civil society, employers, and community leaders in promoting vaccine demand and countering hesitancy.

As outlined in the Immunization Agenda 2030 (IA2030), the life course approach is central to building resilient, equitable, and people-centered immunization systems. By embedding vaccination into all stages of life and across all health touchpoints, countries can transform immunization from a childhood intervention into a cornerstone of lifelong health. This transformation is not only feasible—it is essential to realizing the vision of “health for all” in an increasingly interconnected and risk-prone world. The decisions made today will determine whether future generations can live longer, healthier lives protected by the full benefits of immunization. This is not only a path to improved health outcomes but also a critical pillar of universal health coverage and pandemic preparedness.

### 4.4. Strengthening Health Systems and Cold Chain Infrastructure

As the world prepares for the future of immunization, the next 50 years will require transformative investments in health systems and cold chain infrastructure to ensure equitable, timely, and efficient vaccine delivery. The growing complexity of immunization programs—driven by life course vaccination platforms, new vaccine technologies, and the need for pandemic preparedness—demands a robust, resilient, and adaptive health system backbone.

At the center of this transformation is the cold chain. The cold chain ensures vaccines remain potent from manufacture to administration. However, cold chain systems in many low- and middle-income countries (LMICs) remain fragile, constrained by unreliable electricity, insufficient maintenance, and lack of real-time monitoring. Innovations are bridging these gaps. Solar direct drive (SDD) refrigeration systems now provide sustainable, off-grid cooling solutions. Since 2017, UNICEF and partners have deployed over 180,000 refrigerators, and more than half (100,000) are solar-powered, to enhance cold storage capacity in hard-to-reach settings. These systems reduce reliance on fuel-based generators and improve vaccine availability even in areas without grid power.

To further optimize vaccine potency and reduce wastage, heat-stable and Controlled Temperature Chain (CTC)-qualified vaccines are being introduced [42]. Vaccines such as MenAfriVac (meningitis A), HPV, typhoid, and cholera now tolerate controlled excursions outside the traditional 2–8 °C range, easing logistical challenges and enabling last-mile delivery to remote populations. These innovations are particularly crucial for campaigns, outreach services, and emergency response where refrigeration is limited or absent.

The WHO’s PQS initiative is advancing immunization logistics by introducing Equipment Monitoring System (EMS) specifications that enable real-time monitoring of cold chain performance [43]. These standards enhance interoperability with electronic LMIS platforms, improving asset management, maintenance, and overall reliability and sustainability of vaccine delivery systems.

Active vaccine carriers [44] are enhancing immunization reach by maintaining cold temperatures during long outreach sessions, enabling safe vaccine delivery in remote areas. Simultaneously, green waste management technologies [45]—such as steam sterilization, microwave treatment, and plasma gasification—are reducing the environmental impact of vaccine waste. Together with EMS-enabled cold chain monitoring, these innovations are strengthening immunization systems to be more reliable, equitable, and sustainable globally.

New product innovations are further streamlining logistics and improving user acceptability. Microarray patches (MAPs), or microneedle patches, represent one of the most promising breakthroughs in vaccine delivery [33]. These needle-free patches deliver dry-form vaccines painlessly into the skin and offer the following multiple advantages: they are heat-stable, easy to administer by non-health professionals, and eliminate risks of needle-related injuries or contamination. For measles and rubella, MAPs are in advanced clinical development and have demonstrated strong immunogenicity and programmatic feasibility in early trials. Because they do not require reconstitution or cold-chain-intensive storage, MAPs have the potential to revolutionize outbreak response and routine immunization in resource-limited settings [33,46].

Additionally, innovations in packaging and monitoring—like vaccine vial monitors (VVMs) and threshold indicators—are empowering frontline workers to verify vaccine exposure to heat. Freeze-preventive carriers are addressing the often-overlooked threat of freezing during transport, a major cause of potency loss for freeze-sensitive vaccines.

Digital technology is another critical enabler. Electronic Logistics Management Information Systems (eLMIS), Electronic Immunization Registries (EIRs), and real-time temperature monitoring systems are revolutionizing supply chain visibility. For example, India’s Electronic Vaccine Intelligence Network (eVIN) has been instrumental in reducing stockouts and improving cold chain equipment performance, resulting in higher coverage and reduced wastage [47]. In Zambia and Tanzania, EIRs have improved data accuracy, immunization tracking, and the identification of zero-dose children.

Looking ahead, future-proofing immunization will also depend on integrating cold chains and logistics within broader health systems. This means aligning with primary health care (PHC), ensuring cross-program efficiencies, and linking vaccine delivery to civil registration and vital statistics (CRVS) systems for better targeting and accountability. Moreover, embracing drone delivery, automated stock management, and regional manufacturing of cold chain equipment can ensure timely and locally relevant solutions.

The success of immunization programs heavily depends on the strength and availability of a well-trained health workforce, particularly vaccinators. A global shortage of health care personnel—especially in rural and underserved areas—hinders effective vaccine delivery. Community health workers (CHWs) play a crucial role in bridging this gap, with evidence showing they administered vaccines in 20 of 75 countries with CHW programs [48]. Despite their potential, CHWs often face challenges such as insufficient training, support, and supervision. Strengthening the health workforce is essential to enhance the reach and effectiveness of immunization programs, ultimately improving public health outcomes. In conclusion, the next 50 years of immunization hinge on building resilient, smart, and sustainable health systems that can support increasingly complex vaccine demands. This includes leveraging innovation, local capacity building, public–private partnerships, and data-driven management to create a future where no one is left behind due to cold chain failures or system inefficiencies.

### 4.5. Ensuring Sustainable Financing for Immunization Programs

Securing sustainable financing is one of the most critical challenges and imperatives for the future of global immunization. The estimated cost of fully vaccinating a child [49] through the second year of life, based on standard immunization schedules, is approximately USD 73.15. While investments in immunization in low- and middle-income countries (LMICs) surpassed USD 112 billion from 2000 to 2017 [50], financial inefficiencies and systemic weaknesses persist, hampering progress toward equitable coverage and long-term program resilience. These challenges have been exacerbated by the COVID-19 pandemic, which, while catalyzing additional investment—such as Gavi’s cumulative support of over USD 23 billion to LMICs—also revealed the fragility of immunization systems overly reliant on external aid and unable to absorb and manage funds efficiently [51].

Across 94 low- and middle-income countries, the total projected cost of delivering 10 key vaccines from 2011 to 2030 is USD 70.8 billion under a base case scenario [52]. However, there remains a significant funding gap of USD 38.4 billion, largely driven by the cost of immunization service delivery, which accounts for 86% of the shortfall [50]. One of the main issues lies in the discrepancy between budget allocations and actual expenditures, often driven by weak public financial management (PFM) systems [53]. Underutilized budgets, procurement bottlenecks, and lack of accountability result in missed opportunities to reach children with life-saving vaccines. In many LMICs, declining tax revenues, fragmented donor assistance, and the unreliability of health insurance contributions in informal economies have placed additional pressure on already constrained health budgets. These fiscal vulnerabilities underscore the need to shift from short-term donor dependency to long-term domestic sustainability.

To chart a more resilient course, governments and partners must adopt a comprehensive strategy that blends innovation, domestic resource mobilization, and enhanced system efficiency. Innovative financing instruments such as vaccine bonds—like the USD 1 billion raised by the International Finance Facility for Immunization (IFFIm) in 2024 [54]—demonstrate the potential for rapidly converting donor pledges into immediate cash to support vaccine delivery in LICs. Meanwhile, Gavi’s co-financing model has successfully encouraged countries such as India and Angola to progressively assume greater responsibility for their immunization budgets, fostering national ownership and sustainability.

Strengthening domestic financing is crucial but must be matched with improved PFM capacity. Digitalization of financial systems—including the use of integrated financial management information systems (IFMIS), real-time dashboards, and electronic procurement platforms—can enhance transparency, streamline budget execution, and improve expenditure tracking [55]. These tools help close the loop between planning, resource allocation, and delivery of immunization services, reducing delays and corruption risks.

In addition, expanding fiscal space for health can be achieved through multiple avenues: broadening the domestic tax base; earmarking “sin taxes” on tobacco, alcohol, and sugary drinks; leveraging debt relief for health investment; and exploring blended financing approaches, including insurance schemes and philanthropic contributions. Market-shaping tools such as pooled procurement (e.g., via the UNICEF Supply Division or PAHO Revolving Fund) and advanced market commitments can also lower vaccine costs and ensure supply stability [56].

However, financial sustainability cannot be achieved in isolation. It requires coordinated, whole-of-government action, where ministries of health and finance, civil society, private sector actors, and communities work together to ensure efficient and equitable use of resources. This also means aligning donor assistance with national systems, promoting flexible, predictable, and context-specific funding modalities that facilitate transition to domestic financing without compromising program continuity.

Since human resources, service delivery, supply chains, and capital costs are the primary cost drivers of immunization delivery, countries should consider integrating vaccination programs with primary health care systems to reduce overall program costs significantly [57]. Looking ahead, sustainable immunization financing must become a core pillar of resilient health systems. This will require bold political commitment, strategic investments in local capacity, and a deliberate focus on maximizing value for money. Only through such an integrated and forward-looking approach can the global community ensure that the promise of immunization—saving millions of lives each year—remains a reality for generations to come.

To ensure sustainable and equitable immunization financing, a whole-of-government approach involving health and finance ministries, civil society, and communities is essential, as it focuses on maximizing impact in resource-constrained environments.

## 5. Conclusions: Renewing the Global Commitment

The success of the Expanded Programme on Immunization over the past 50 years stands as one of the greatest public health triumphs in history. However, challenges such as vaccine hesitancy, pandemic-related disruptions, and equitable access remain formidable.

Looking ahead, immunization efforts must expand beyond childhood vaccination, embrace technological advancements, and strengthen health systems to ensure that every person, regardless of their circumstances, has access to life-saving vaccines.

With continued global commitment, funding, and innovation, immunization will remain a pillar of disease prevention and public health. The future of vaccination is not just about new technologies but about ensuring that every individual—regardless of where they live—has access to life-saving vaccines.

This Special Issue, called “50 Years of Immunization—Steps Forward”, covers over 25 articles and offers historical perspectives as well as the state of EPI today. It includes articles with global scope, LMIC scope, regional-specific, and country-specific case studies. It covers several vaccine-specific articles, including measles, polio, HPV, cholera, dengue, influenza, zoster, hepatitis E, shigella, and pertussis.

As we celebrate the legacy of EPI, we must recommit to protecting future generations from preventable diseases since immunization is not just about vaccines or an investment in health; it is an investment in equity, humanity, and the survival of future generations, and the right to a healthier life for all.

## Figures and Tables

**Table 1:** Key achievements of EPI over 50 years.

Dramatic Improvements in Child Survival Rates
Immunization contributed to a 40% decline in global under-five mortality from 1974 to 2024. Pneumococcal and rotavirus vaccines have significantly reduced hospitalizations due to pneumonia and diarrhea. Countries with high routine vaccination coverage saw improved overall health indicators.
**Disease Eradication and Reduction in Vaccine-Preventable Diseases**
○ Smallpox was declared eradicated in 1980, proving the success of mass vaccination campaigns. ○ Measles deaths declined by 73% between 2000 and 2018 due to routine immunization and mass vaccination campaigns. ○ Polio cases dropped by over 99.9%, with wild poliovirus cornered in a few neighboring districts of two endemic countries. Neonatal tetanus has been eliminated in most countries, with 10 remaining, reducing newborn mortality significantly.
**Global Immunization Reach**
○ Today, over 85% of infants worldwide receive at least three doses of the DPT vaccine. Hardly any other health intervention reached as many children as possible each year, even considering a growing cohort of births, in particular in Africa. ○ Immunization programs now reach the most remote and marginalized communities, including refugee camps and conflict zones, and are now expanded to target adolescents and adults, including HPV, influenza, and COVID-19 vaccines.
**Development and Deployment of New Vaccines**
○ The introduction of HPV vaccines for adolescent girls has cut cervical cancer risk by over 80% in some countries. ○ The development of malaria and Ebola vaccines represents a historic milestone in immunization science. ○ Regional vaccines such as Japanese encephalitis, TCV, etc.
**Key Innovations in Cold Chain Logistics**
○ Vaccine Vial Monitors (VVMs): These small labels change color if a vaccine is exposed to heat for too long, reducing vaccine wastage. ○ Solar-Powered Refrigerators: A game-changer for off-grid and rural areas, ensuring continuous refrigeration without reliance on electricity. ○ Ultra-Cold Storage Solutions for mRNA Vaccines: The development of portable ultra-cold freezers has expanded mRNA vaccine distribution to low-resource settings. ○ Remote Temperature Monitoring (RTM) Systems: Digital tracking devices now send real-time alerts if vaccines are exposed to unsafe temperatures. ○ Next-Generation Cold Chain Equipment (CCE): Introduction of passive cold chain devices that can store vaccines for up to 30 days 5–7 days without electricity.

## Abbreviations

The following abbreviations are used in this manuscript:

BCU	big catch-up
EPI	Essential Programme on Immunization, previously called Expanded Programme on Immunization
GAVI	Global Alliance Vaccine Initiative
GPEI	Global Polio Eradication Initiative
IA2030	Immunization Agenda 2030
RI	routine immunization
ZD	zero dose
UI	under-immunized
